# A case of anti-laminin 332-type mucous membrane pemphigoid mimicking linear IgA bullous dermatosis

**DOI:** 10.1016/j.jdcr.2026.06.037

**Published:** 2026-06-20

**Authors:** Xinyu Du, Jingyang Dang, Jiahui Zhao, Panpan Shang

**Affiliations:** a Department of Dermatology and Venereology, Peking University First Hospital, Beijing, China; b National Clinical Research Center for Skin and Sexually Transmitted Diseases, Peking University First Hospital, Beijing, China

**Keywords:** bullous disease, corticosteroids, immunoglobulin, laminin

## Case report

**Fig 1:**
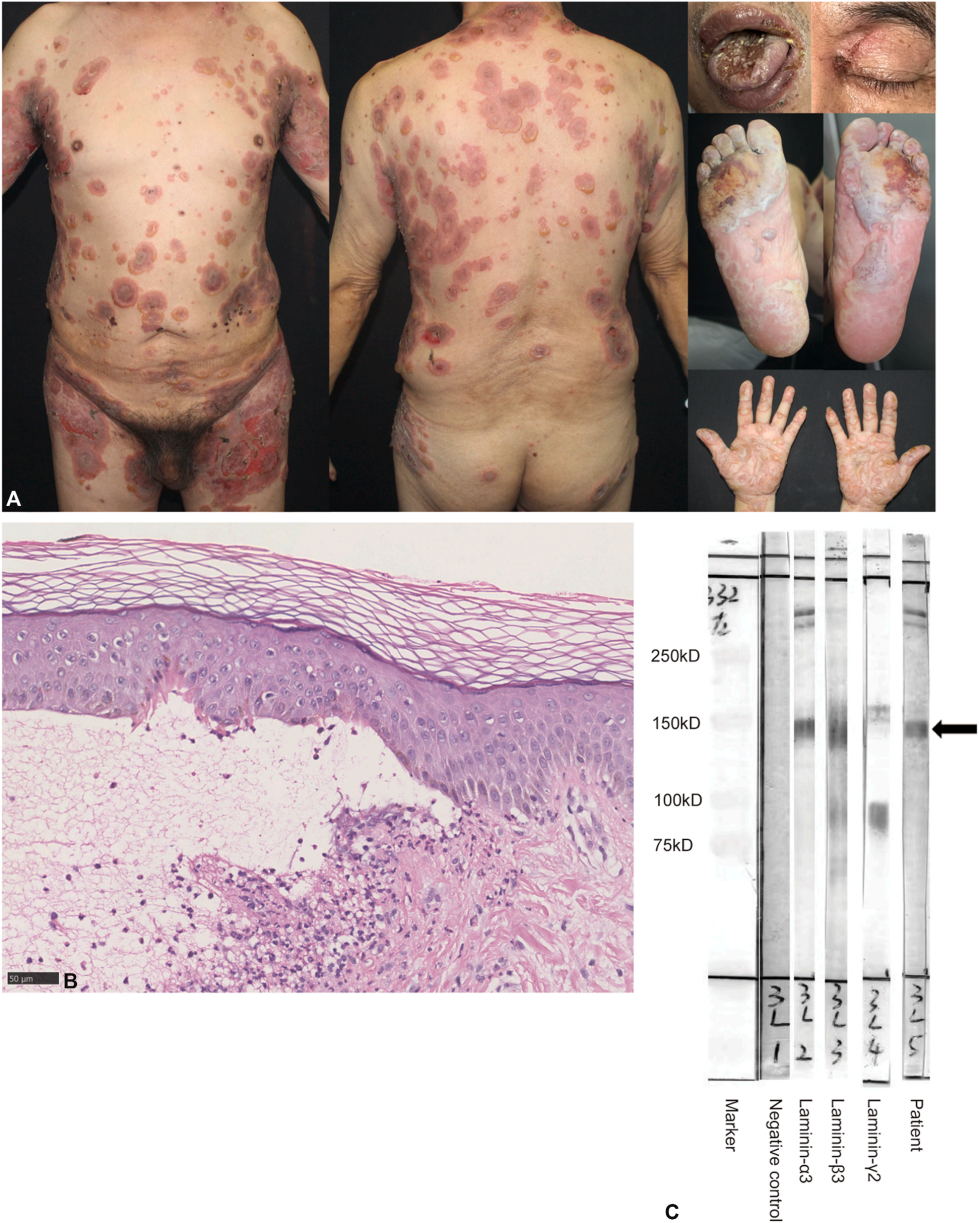
**A,** Clinical presentation showing widespread erythematous plaques and tense bullae on the trunk, extremities, groins, palms, and soles. Oral and nasal mucosal erosions with hemorrhagic crusting on the lips and eyelids were also noted. **B,** Histopathological examination of lesional skin demonstrating subepidermal blister formation with dermal inflammatory infiltration. **C,** Immunoblotting revealed IgG reactivity against the α-3 subunit (*arrow*), confirming the diagnosis of laminin-332-type mucous membrane pemphigoid.

A 68-year-old man presented with erythematous lesions and tense bullae on the trunk and extremities, accompanied by oral mucosal erosions, after he suffered from a flu-like illness marked by high fever and sore throat. The patient denied any recent medication use, including antibiotics or nonsteroidal anti-inflammatory drugs. Upon referral to our hospital, physical examination revealed erosion of the oral and nasal mucosa, with hemorrhagic crusting on the lips and eyelids, and conjunctival involvement was absent. Annular erythematous plaques and bullae were widely distributed on the trunk, extremities, axillae, groins, palms, and soles (Fig 1, *A*). Histopathologic examination demonstrated subepidermal bullae (Fig 1, *B*), a mixed inflammatory infiltrate composed of lymphocytes and neutrophils, with scattered eosinophils.

**Question 1: Based on the clinical presentation and histopathologic findings, what is the most likely diagnosis?**

**A.** Erythema multiforme
**B.** Bullous pemphigoid
**C.** Pemphigus vulgaris
**D.** Mucous membrane pemphigoid
**E.** Linear IgA bullous dermatosis

**Answer:**

**The correct answer is D, mucous membrane pemphigoid.**

Serum enzyme-linked immunosorbent assay results for anti-BP180 and anti-BP230 IgG were negative. Direct immunofluorescence revealed linear IgG deposition along the basement membrane zone, whereas IgA deposition was absent. Indirect immunofluorescence and salt-split indirect immunofluorescence results were negative. Immunoblotting using purified laminin-332 identified IgG reactivity against the α3 subunit (Fig 1, *C*). Based on these findings, a diagnosis of anti-laminin 332-type mucous membrane pemphigoid (MMP) was established.

## Discussion

MMP is a rare autoimmune subepidermal blistering disorder that predominantly involves mucosal surfaces, whereas cutaneous lesions are usually limited and less extensive.^1^ Classic MMP is characterized by chronic mucosal erosions that may heal with scarring, particularly in ocular disease. In contrast, our patient exhibited unusually widespread cutaneous involvement with prominent annular erythematous plaques and tense bullae affecting the trunk, extremities, palms, and soles. Severe palmoplantar involvement is uncommon in typical MMP. A previously reported case of anti-laminin-332 MMP demonstrated similarly extensive skin lesions,^2^ suggesting that widespread cutaneous manifestations may represent a clinical feature of this subtype. Additionally, although mucosal lesions were present in our patient, no scarring developed during follow-up, which further distinguishes this presentation from classic cicatricial MMP.

Clinically, this case closely resembled linear IgA bullous dermatosis because of the widespread annular plaques, tense bullae, and mucosal involvement.^3^ Histopathology demonstrated subepidermal blistering with numerous neutrophils in the papillary dermis, which may overlap with the histologic appearance of linear IgA bullous dermatosis. However, direct immunofluorescence revealed linear IgG deposition along the basement membrane zone with complete absence of IgA, effectively excluding linear IgA bullous dermatosis.

Several findings also initially suggested bullous pemphigoid, including advanced age, subepidermal blistering, eosinophils in the papillary dermis, and linear IgG deposition.^4^ Nevertheless, prominent mucosal involvement, negative enzyme-linked immunosorbent assay results for anti-BP180 and anti-BP230 antibodies, negative salt-split indirect immunofluorescence, and immunoblot confirmation of IgG reactivity against laminin-332 α3 established the diagnosis of anti-laminin-332–type MMP.

Most reported cases respond to systemic corticosteroids at low-to-moderate doses, with or without adjunctive immunosuppressants.^5^ However, our patient demonstrated disease progression despite standard-dose corticosteroids and dupilumab. Following corticosteroid pulse therapy, the patient improved rapidly and remained relapse-free during 2 years of follow-up.

## Conflicts of interest

None disclosed.
